# Prediction and course of symptoms and lung function around an exacerbation in chronic obstructive pulmonary disease

**DOI:** 10.1186/1465-9921-13-44

**Published:** 2012-06-06

**Authors:** Maarten van den Berge, Wim CJ Hop, Thys van der Molen, Jan A van Noord, Jacques PHM Creemers, Ad JM Schreurs, Emiel FM Wouters, Dirkje S Postma

**Affiliations:** 1 Department of Respiratory Medicine, University Medical Center Groningen, GRIAC Research Institute, University of Groningen, Groningen, The Netherlands; 2 Department of Biostatistics, Erasmus Medical Center Rotterdam, Rotterdam, The Netherlands; 3 Department of General Practice, University Medical Center Groningen, GRIAC research Institute, University of Groningen, Groningen, The Netherlands; 4 Atrium Medisch Centrum, Heerlen, The Netherlands; 5 Catharina Hospital Eindhoven, Eindhoven, The Netherlands; 6 Bosch Medisch Centrum, ‘s-Hertogenbosch, Hertogenbosch, The Netherlands; 7 Department of Respiratory Medicine, University Hospital Maastricht, Maastricht, The Netherlands; 8 Department of Pulmonology, University Medical Center Groningen, University of Groningen, 9713, Groningen, GZ, The Netherlands

**Keywords:** COPD, Exacerbations, CCQ, Symptoms

## Abstract

**Background:**

Frequent exacerbations induce a high burden to Chronic Obstructive Pulmonary Disease (COPD). We investigated the course of exacerbations in the published COSMIC study that investigated the effects of 1-year withdrawal of fluticasone after a 3-month run-in treatment period with salmeterol/fluticasone in patients with COPD*.*

**Methods:**

In 373 patients, we evaluated diary cards for symptoms, Peak Expiratory Flow (PEF), and salbutamol use and assessed their course during exacerbations.

**Results:**

There were 492 exacerbations in 224 patients. The level of symptoms of cough, sputum, dyspnea and nocturnal awakening steadily increased from 2 weeks prior to exacerbation, with a sharp rise during the last week. Symptoms of cough, sputum, and dyspnea reverted to baseline values at different rates (after 4, 4, and 7 weeks respectively), whereas symptoms of nocturnal awakening were still increased after eight weeks. The course of symptoms was similar around a first and second exacerbation. Increases in symptoms and salbutamol use and decreases in PEF were associated with a higher risk to develop an exacerbation, but with moderate predictive values, the areas under the receiver operating curves ranging from 0.63 to 0.70.

**Conclusions:**

Exacerbations of COPD are associated with increased symptoms that persist for weeks and the course is very similar between a first and second exacerbation. COPD exacerbations are preceded by increased symptoms and salbutamol use and lower PEF, yet predictive values are too low to warrant daily use in clinical practice.

## Introduction

Goals in the management COPD are to prevent and control symptoms, to reduce the frequency and severity of exacerbations, and to improve health status and exercise tolerance and prevent disease progression [1]. In particular, frequent exacerbations induce a high economic, social, and personal burden. Exacerbations are regarded as important to disease prognosis, since an increased frequency of these episodes has been reported to be associated with accelerated lung function decline and increased mortality rates, particularly if these require admission to hospital [2-4].

Studies in asthma have shown that exacerbations resolve generally within 2 weeks with respect to symptoms, salbutamol use and peak expiratory flow (PEF) values [5]. Data on exacerbations in COPD are still rather scarce [6-9], one study suggesting that symptoms resolve within two weeks just as in asthma [7], though another post hoc analysis in a large group of COPD patients suggests that this would take longer [10]. A better understanding of the course of symptoms around COPD exacerbations is important. It may help to optimize the duration of treatment and follow-up after a COPD exacerbation. In addition, the recognition of symptoms and signs that predict a pending COPD exacerbation will enable physicians to start treatment in an early phase.

According to the GOLD (Global Obstructive Lung Disease) guidelines, the goals of clinical control in patients with COPD include both health status (improved exercise tolerance and emotional function) and clinical goals (prevention of disease progression and minimization of symptoms). The Clinical COPD questionnaire (CCQ) is the first practical instrument to be used for routine evaluation of clinical control concerning patients with COPD in general practice [11,12]. A worse health status may contribute to hospitalisations [13], yet this has not been reported for CCQ so far.

In a previous report on the COSMIC (COPD and Seretide: a Multi-Center Intervention and Characterization) population with moderate to severe COPD, it has been shown that there are no significant differences with respect to the number of moderate to severe exacerbations experienced by patients in whom inhaled steroids were withdrawn after a run-in period with fixed dose of fluticasone (500 μg) and salmeterol (50 μg), versus those who continued this treatment [14]. Only the number of mild exacerbations (defined as increased need for rescue medication above the run-in period) was significantly larger in the group in which inhaled steroids were withdrawn.

The current report explores which factors determine the course of moderate and severe exacerbations, i.e. the duration and resolution of an exacerbation in relation to symptoms, additional use of beta-agonists and peak expiratory flow (PEF) in this group of COPD patients. It furthermore assesses the similarity of the course of symptoms around a first and second exacerbation. Information on the intra-individual variability of the course of symptoms around an exacerbation is important, since we wished to assess whether a change in symptoms can predict the occurrence of a moderate or a severe COPD exacerbation.

## Methods

### Patients and study design

Inclusion and exclusion criteria, methods for lung function measurements and design of the COSMIC study have been reported previously [14]. In short, all patients were diagnosed with COPD according to the current GOLD guidelines and were selected based on the occurrence of at least 2 exacerbations in the year preceding entry to the study [1]. During a three-month run-in period, all patients received combined salmeterol 50 μg and fluticasone 500 μg (Seretide® or Advair® 50/500 μg) twice daily. Thereafter, patients were randomized to a 12-month treatment with either salmeterol/fluticasone or salmeterol alone [14]. We obtained approval from all ethics committees at each participating site, and all patients provided written informed consent.

### Exacerbations and rescue medication use

Patients recorded use of rescue medication (salbutamol) over the last 24 hours in daily paper record cards. If the patient’s condition worsened and a course of oral corticosteroids (standardized course of prednisolone tablets 30 mg/day for 10 days; at the discretion of the physician accompanied by a 10-day course of antibiotics) was indicated based on the judgment of the research physician or general practitioner, the exacerbation was defined as moderate. If hospitalization was required at the discretion of the clinician, the exacerbation was considered severe. The time of onset and end of exacerbation was based on clinical judgment according to the research physician at each site.

### Daily record cards and morning PEF

Every morning patients recorded severity scores of symptoms over the previous 24 hours on shortness of breath, cough, sputum production and night-time sleep disturbance (breathlessness 0 (none) to 4 (breathless at rest); cough and sputum production 0 (none) to 3 (severe); night-time sleep disturbance due to respiratory symptoms 0 (none) to 4 (did not sleep at all). Patients recorded their daily morning PEF throughout the run-in and the treatment period, prior to the inhalation of COPD (study) medication. The highest of three values was used for analysis.

### Health status

The Clinical COPD Questionnaire (CCQ) was administered to assess health status, a higher score meaning greater impairment. It is a 10-item, self administered questionnaire that can be completed in less than 2 minutes. Items are divided into three domains: symptom, functional state and mental state. Patients are required to respond to each item on a seven-point Likert scale where 0= asymptomatic/no limitation and 6 = extremely symptomatic/total limitation. The final score in the mean of all ten items and scores for the three domains can be calculated separately if required.

### Statistical analysis

Poisson-regression was used to evaluate the relation between the annual exacerbation rate and the factors FEV_1_ (%predicted), age, gender, smoking status and CCQ. Mixed model Anova (SAS PROC MIXED) was used to evaluate associations between these factors and the log-transformed durations of both the moderate and severe exacerbations. Profiles of all longitudinal diary card data were used to characterize exacerbations. Only patients with a proper scoring of diaries in each of both periods were evaluated in these analyses, i.e. at least 50 percent of diary days. In addition patients were required not to have a new exacerbation in the 8-week period after the onset of the exacerbation. We analyzed the diary card data to investigate whether the increase in total or separate symptom scores could predict the occurrence of an exacerbation at each day of follow-up as follows. The increase of the mean symptom score was calculated for each patient separately during the week before the day of onset of the exacerbation, i.e. days 1 to −6, as compared to the mean symptom score during the week covering days −8 to −14. These increases were calculated for all patients during each day of follow-up, irrespective of whether the patient had experienced an exacerbation or not. So in fact a time-window of 14 days width of 2 successive weeks is used and this window is moved one day along time step by step, resulting at each step in a difference of 2 moving averages of symptom scores. For example, if a patient has a mean score of 10 points during days 101 to 107 and this was 13 points during days 108 to 114, then the value of the calculated increase at day 115 equals +3 points. The information at all follow-up days can be combined using Cox-regression, with the calculated increases of symptom scores per patient at each day of follow-up as a time-dependent covariate. With this method, the daily risk of an exacerbation is quantified in relation to the observed longitudinal changes in symptom scores. Regarding prediction of an exacerbation, only data to the first exacerbation were analyzed. The same method with moving averages was used to investigate the predictive value of changes in daily PEF measurements and salbutamol use. Receiver operating characteristic curves (ROC-curves) were constructed to evaluate the predictive value of the changes in total or separate symptom scores, PEF or salbutamol use as explained above. An area under the Receiver Operating Curve (AUC) of 0.8 or greater is generally considered to indicate a good predictor. A two-sided p-value below 0.05 was considered to be significant in all analyses.

## Results

Baseline characteristics of the 373 patients enrolled are presented in Table 1. Follow-up in the salmeterol and salmeterol/fluticasone group amounted to 161 and 179 person-years respectively. During this period, 109 patients in the salmeterol group experienced a total of 254 exacerbations, 19 being severe. In the salmeterol/fluticasone group, 115 patients had a total of 238 exacerbations, 24 being severe. Poisson regression analysis, allowing for treatment, gender, age, baseline FEV_1_ % predicted and smoking status, showed that the annual moderate to severe exacerbation rate was 1.6 per patient year in the salmeterol group and 1.3 per patient year in the salmeterol/fluticasone group (1.2-fold greater with salmeterol, 95% confidence interval ((CI): 0.9-1.5; p=0.15). When considering moderate and severe exacerbations separately, no significant difference was observed between the treatment arms as well.

**Table 1:** Demographic data and baseline characteristics of randomized patients

	**Salmeterol n=184**		**Salmeterol + Fluticasone n=189**	
Age, years	64.0	(7.7)	63.0	(7.9)
Male	137	(75)	138	(73)
Current smoker	64	(35)	74	(39)
Pack-years smoked	37.8	(18.1)	34.8	(17.4)
Prebronchodilator FEV_1_ % predicted*	48.2	(12.9)	47.4	(13.9)
Reversibility, % predicted FEV_1_*	4.9	(4.1)	4.7	(3.5)
CCQ Total score *	1.7	(0.9)	1.6	(0.8)
CCQ functional	1.7	(1.1)	1.7	(1.0)
CCQ emotional	0.5	(0.9)	0.5	(0.8)
CCQ symptoms	2.3	(1.1)	2.1	(0.9)

* at randomization. FEV_1_: forced expiratory volume in one second. Data are presented as number (%) or mean (SD). CCQ= Control of COPD Questionnaire; Reversibility was assessed by inhaling 400μg salbutamol using a Volumatic® spacer.

### Duration of exacerbation

There were 492 moderate or severe exacerbations in a total of 224 COPD patients, median duration of these 492 exacerbations being 10 days (range 2–119 days). The duration of a severe exacerbation was on average 46% longer than a moderate one (geometric mean 18.6 and 11.5 days respectively, p=0.001); no significant differences existed between the treatment arms. Mixed model Anova, allowing for gender, age, current and pack-years smoking, treatment and FEV_1_ % predicted, showed that the number of pack-years smoking was the only significant predictor for the duration of a severe exacerbation. With every 5 pack-years smoking, the mean duration of a severe exacerbation increased 1.15 times. There were no significant effects of any of these parameters on the duration of a moderate exacerbation. Furthermore, the CCQ total score at baseline did not significantly contribute to the duration of moderate and/or severe exacerbations. The same applied to the separate domains of CCQ.

### Course of symptoms, PEF and salbutamol use before and after the first moderate or severe exacerbation

**Figure 1:**
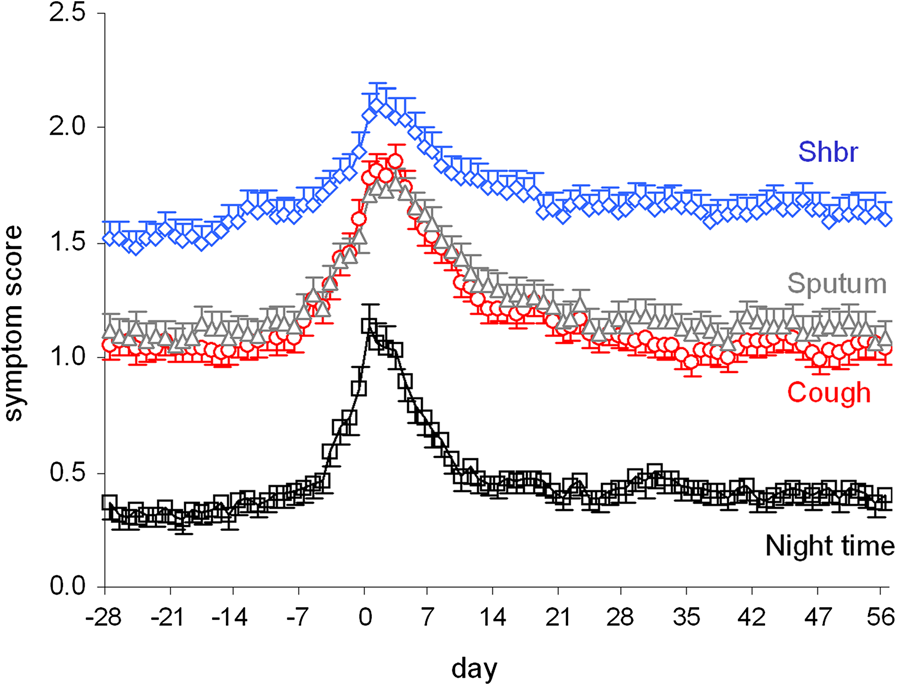
**Mean symptom scores (with SEM) from diary for shortness of breath (shbr), (scale 0–4), sputum (scale 0–3), cough (scale 0–3) and night-time sleep disturbance (scale 0–4) according to day number before and after the onset (day 0) of the first exacerbation.** Evaluated patients had to contribute at least 50% of properly scored diary days in the 4 weeks period before the onset. The same applied to the 8 weeks period after the onset. In addition no new exacerbations had to occur within the latter 8 weeks period, leaving n=137 patients for analysis for all curves. Recovery from an exacerbation is slowest with shortness of breath, followed by nocturnal respiratory symptoms, sputum, and cough.

Of the 224 patients who experienced an exacerbation, 48 were labeled as treatment failures and excluded from further analyses as they suffered from a new exacerbation within 8 weeks after the onset of the first exacerbation. Of the remaining 176 patients, 39 were excluded from analysis because they did not properly fill in their diary cards for more than 50% of the days. Thus, we were able to analyze the course of symptoms around a first moderate or severe exacerbation in 137 patients. Of these 137 patients, 101 were treated with a course of oral prednisolone and antibiotics, whereas 36 patients were treated with oral prednisolone alone. Figure 1 shows the marked similarity in profile of the four different symptom scores. The mean level of symptoms before an exacerbation steadily increased from 2 weeks prior to exacerbation, with a sharp rise during the last week. After the first moderate or severe exacerbation, mean symptom scores were significantly higher than the level during stable disease, i.e. the fourth week before that particular exacerbation. Weekly average symptom scores at two weeks after an exacerbation were generally not back to the average level present during the fourth week prior to that exacerbation. When analyzing the weekly average of salbutamol use and each individual symptom separately, mean salbutamol use and shortness of breath score were still significantly higher during the 6^th^ and 7^th^ week after the exacerbation respectively compared to the weekly average scores during stable disease, i.e. the fourth week prior to the onset of the exacerbation (Table 2). With mean weekly sputum and cough scores, this took 4 weeks, whereas mean weekly night-time awakening scores due to respiratory symptoms lingered on for even longer, i.e. they were still significantly higher than at baseline by 5 weeks with borderline significance thereafter and again a significantly higher score at 8 weeks after an exacerbation. Mean weekly PEF values were back to pre-exacerbation levels within 2 weeks (Table 2). When analyzing the course of symptoms between patients with and without antibiotics, similar results were obtained.

**Table 2:** The average symptoms scores, PEF values and number of puffs of salbutamol use are presented, starting from 4 weeks prior until 8 weeks after the onset of exacerbation

	**Wk-4**	**Wk1**	**Wk2**	**Wk3**	**Wk4**	**Wk5**	**Wk6**	**Wk7**	**Wk8**
Shortness of breath	1.5(.1)	2.0(.1)	1.8(.1)	1.7(.1)	1.7(.1)	1.7(.1)	1.6(.1)	1.6(.1)	1.6(.1)
		*	*	*	*	*	ns	p=.047	ns
Cough	1.1(.1)	1.7(.1)	1.3(.1)	1.2(.1)	1.1(.1)	1.0(.1)	1.0(.1)	1.1(.1)	1.1(.1)
		*	*	*	ns	ns	ns	ns	ns
Sputum	1.1(.1)	1.7(.1)	1.4(.1)	1.2(.1)	1.2(.1)	1.2(.1)	1.1(.1)	1.1(.1)	1.1(.1)
		*	*	*	ns	ns	ns	ns	ns
Nocturnal wakening	0.3(.1)	0.9(.1)	0.5(.1)	0.5(.1)	0.4(.1)	0.5(.1)	0.4(.1)	0.4(.1)	0.4(.1)*
		*	*	*	*	*	ns	p=.06	p=.04
PEF, l/min	279(8)	264(7)	275(8)	276(8)	277(8)	277(8)	278(8)	277(8)	273(7)
		*	ns	ns	ns	ns	ns	ns	ns
Salbutamol , puffs/day	1.5(.2)	2.1(.2)	1.8(.2)	1.7(.2)	1.7(.2)	1.7(.2)	1.8(.2)	1.7(.2)	1.7(.2)
		*	*	*	*	*	*	ns	ns

Data given are mean values, with standard errors between parentheses (repeated measurements Anova estimates). A particular week for a patient was considered evaluable if there were at least 4 valid diary days during that week.

* p<0.05 for the comparison with Wk-4.

### Similarity between a first and second exacerbation

**Figure 2:**
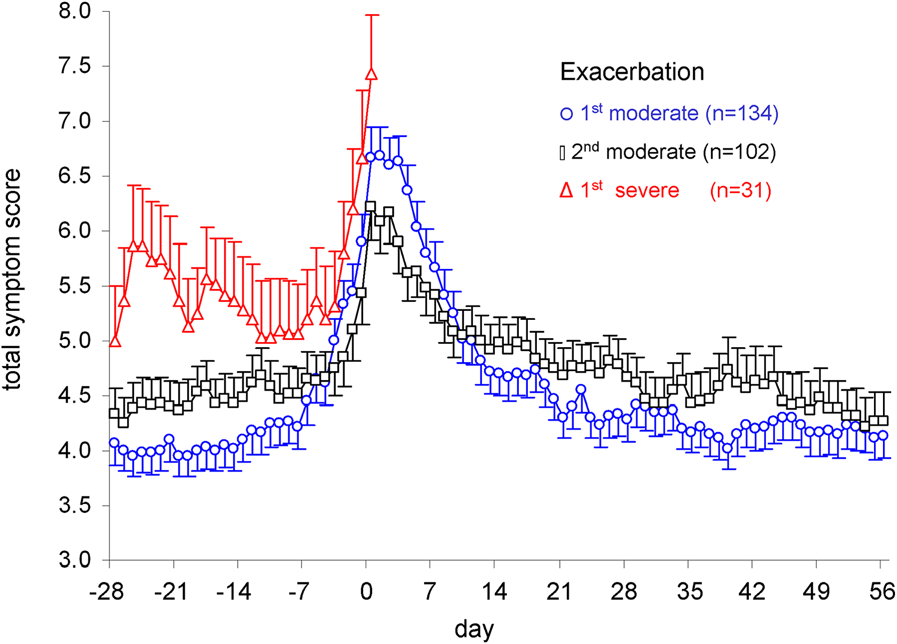
**Mean total symptom scores (with SEM) from diary cards according to day number before (days −28 to 0) and after (days 1 to 56) the onset of an exacerbation.** Day 0 marks the recorded day of the onset of an exacerbation in the clinical record form. Circles, squares and triangles denote the 1^st^ moderate, 2^nd^ moderate and 1^st^ severe exacerbation, respectively. Outcomes for the 1^st^ severe exacerbation are graphed only up to the day of onset because of many missing values after its onset (when patient were admitted to the hospital).

**Figure 3:**
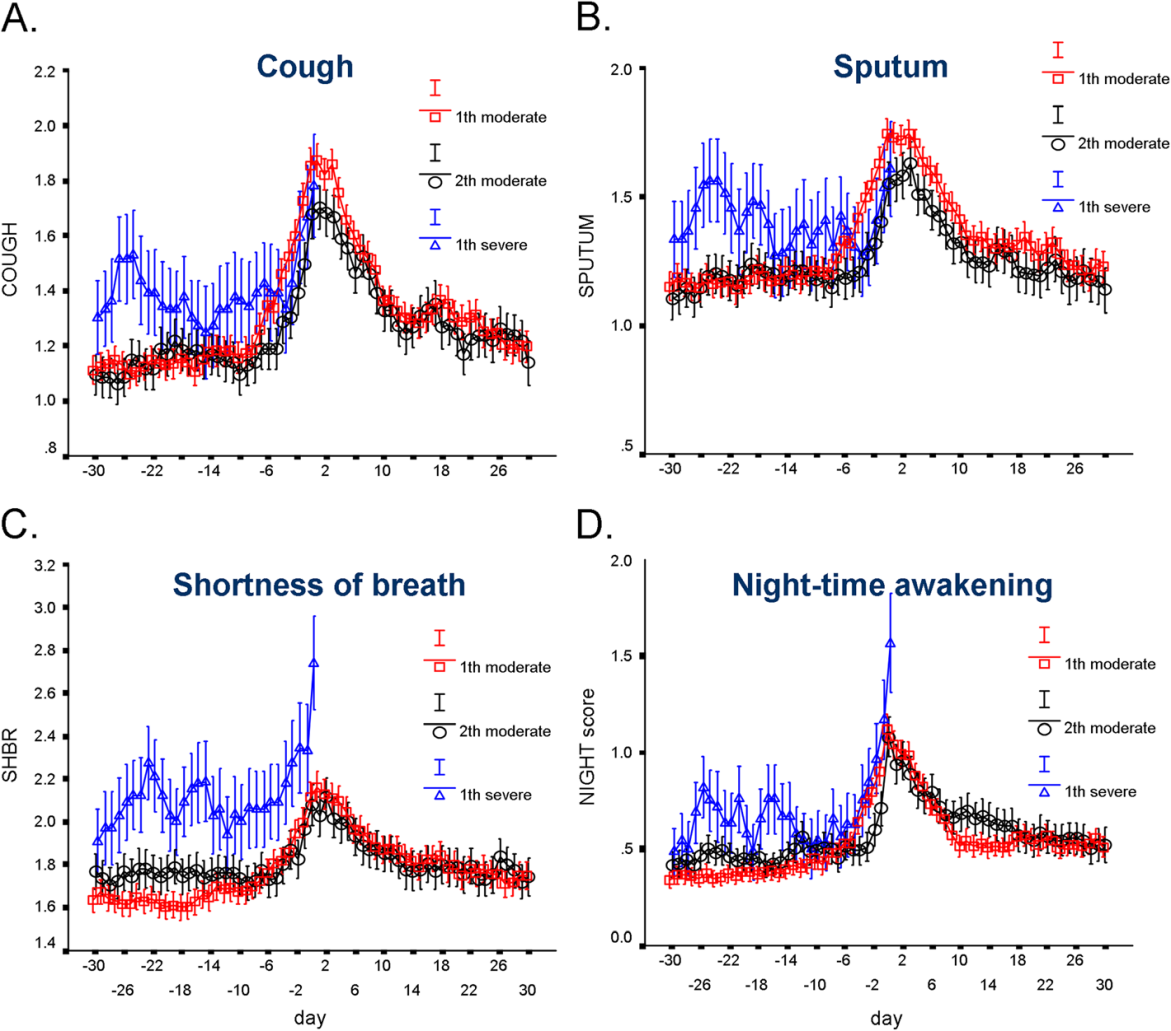
**Mean symptom scores (with SEM) for A) Cough, B) Sputum, C) Shortness of breath, and D) Night-time awakening.** Based on diary cards according to day number before (days −28 to 0) and after (days 1 to 56) the onset of an exacerbation. Day 0 marks the recorded day of the onset of an exacerbation in the clinical record form. Circles, squares and triangles denote the 1^st^ moderate, 2^nd^ moderate and 1^st^ severe exacerbation, respectively. Outcomes for the 1^st^ severe exacerbation are graphed only up to the day of onset because of many missing values after its onset (when patients were admitted to the hospital).

Figure 2 shows that the mean course of the increase and decrease in symptoms was very similar around the onset of a first and second moderate exacerbation. The mean value in the period from day −28 to day 0 did not significantly differ between the 1^st^ and 2^nd^ moderate exacerbation. The same applied to the period from day 1 to day 56 after the start of the exacerbation. In the period before the exacerbation, mean values of total symptom scores were higher for the severe exacerbation as compared to the first and second moderate exacerbations (both p<0.02), the pattern being similar for separate symptoms of cough, sputum, shortness of breath and night-time awakening (Figure 3). Similarly, salbutamol use was significantly higher in the period before the onset of a severe exacerbation and PEF values were significantly lower.

### Prediction of exacerbation by symptoms preceding the first moderate or severe exacerbation

We reported previously [14] that females had a 1.4-fold higher exacerbation rate than males (p=0.03) and that baseline FEV_1_ % predicted strongly associated with the exacerbation rate, i.e. each 10 points lower value of FEV_1_ %predicted led to a 1.2-fold increase in exacerbation rate (p<0.001). Adding baseline CCQ values to this Poisson regression model showed that the CCQ significantly contributed to the exacerbation rate. When adding each domain (i.e. Symptoms, Mental and Functional), the functional domain remained in the Poisson-model with a highly significant contribution (p=0.001). The factor gender became of borderline significance (p=0.08), whereas FEV_1_ % predicted remained significant (p=0.019).

**Figure 4:**
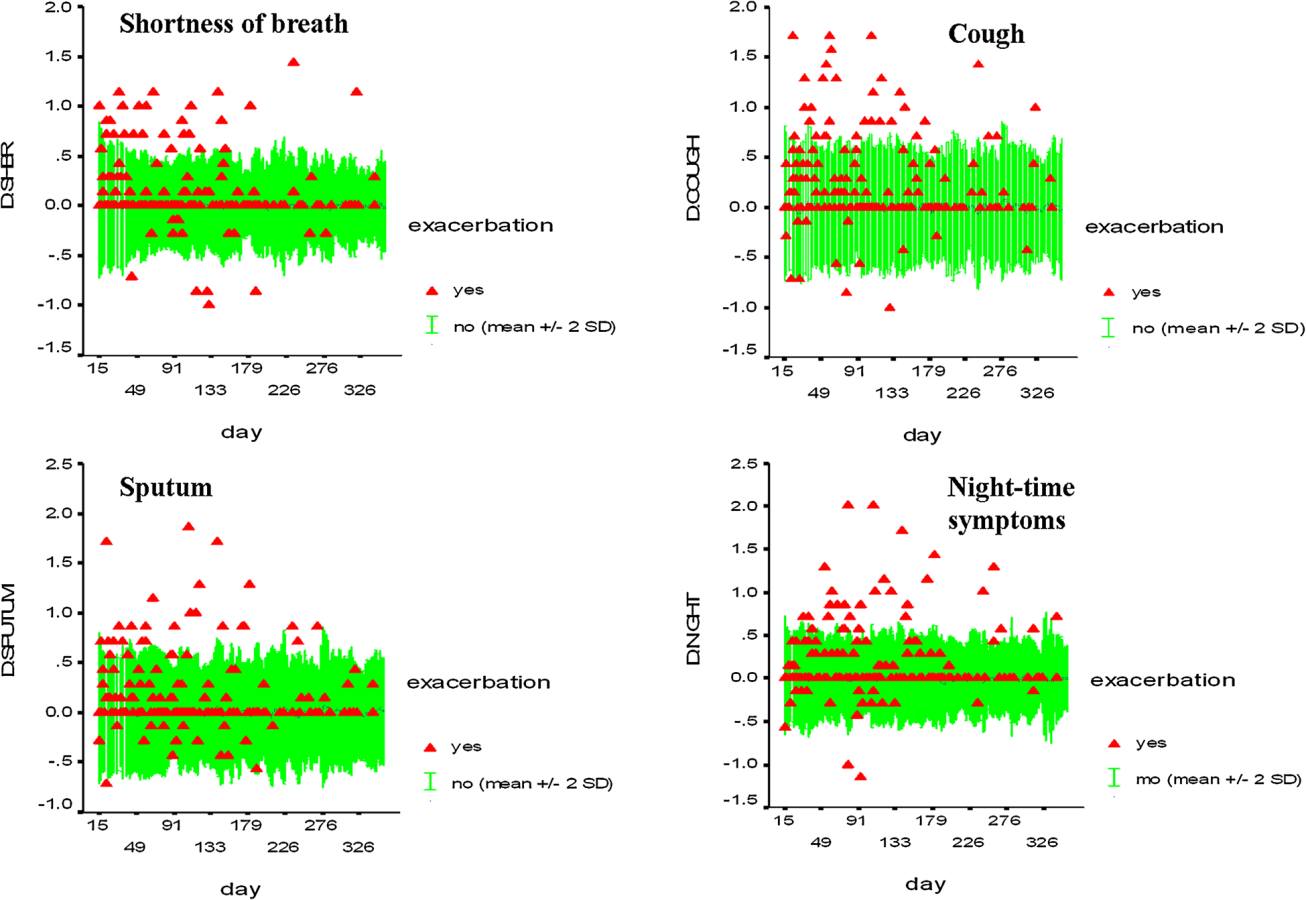
**Graph of increases in moving weekly averages for the different symptom scores from the diaries at each day during follow-up.** Triangles represent individual values of the increase at the day of a patient’s (first) exacerbation. The gray area represents the reference range at each day of follow-up, i.e. mean +/− 2 SD values of the increase for those who did not have an exacerbation at the days indicated.

The averages of the symptom scores in two subsequent weeks were compared to assess whether an exacerbation is pending. Although there were individuals who had a marked increase in symptoms during the week preceding an exacerbation, a large inter-individual variability was observed (Figure 4). Table 3 shows that a change in total symptom score in two subsequent weeks by 1–2 points increased the risk of a moderate to severe exacerbation 4.8 fold, and 8.2 fold when there was an increase of 2–3 points. When assessing the sensitivity and specificity of the change in total symptom score using ROC-curves, the area under the curve (AUC) obtained was 0.70 (Figure 5). AUC values for the separate symptoms were slightly lower: 0.61 for shortness of breath, 0.67 for cough, 0.64 for sputum and 0.63 for nocturnal respiratory symptoms. A similar analysis for the two-week changes in PEF and daily salbutamol use resulted in AUC values of 0.67 and 0.63, respectively. We were unable to perform the above analyses for only severe exacerbations, because of too low numbers.

**Table 3:** Cox-regression analysis of change in average points in total symptoms in two subsequent weeks (maximum score in total symptoms = 14) as risk for a moderate or severe exacerbation

**Change in points**	**Relative Risk (RR)**	**p- value**	**95% Confidence Interval for RR**
<1 point	1^#^	-	
1-2 points	4.8	<0.001	3.1 - 7.5
2-3 points	8.2	<0.001	4.3 – 15.8
>3 points	18.7	<0.001	10.5 – 33.4

# reference category.

**Figure 5:**
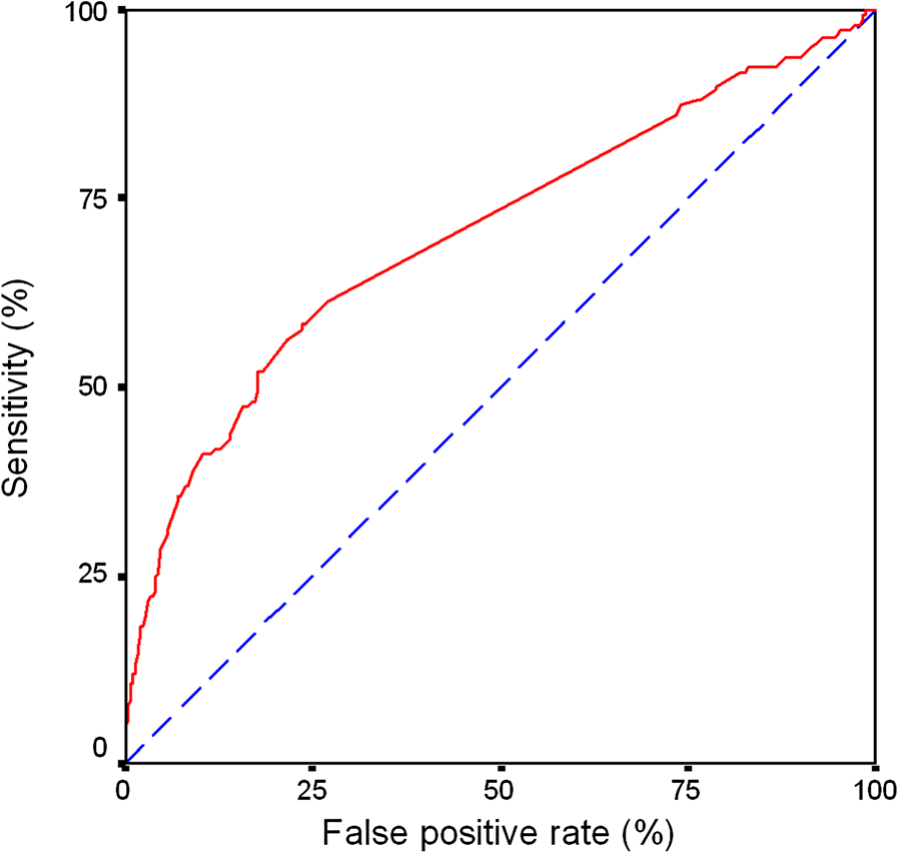
**ROC-curve depicting Sensitivity versus False positive rate, i.e. 1-specificity, of change in total symptom score per week to predict a moderate or severe exacerbation.** Area under curve = 0.70. Dotted diagonal line represents an imaginary test without any predictive value.

## Discussion

Our study shows that exacerbations of COPD are associated with increased symptoms that persist for weeks. Especially shortness of breath and nocturnal awakening due to respiratory symptoms persist for many weeks after the resolution of the acute phase of an exacerbation (7 and 8 weeks respectively). In addition, we demonstrate that the course of symptoms is very similar between a first and second exacerbation. Finally, we show that exacerbations are preceded by lower functional status, increased symptoms and salbutamol use and lower PEF yet predictive values are too low to warrant their use for clinical practice.

This is in contrast to observations in asthma where symptoms during an exacerbation remit within 7–10 days after treatment with prednisone [5]. Especially shortness of breath and nocturnal awakening due to respiratory symptoms persist for many weeks after the resolution of the acute phase of an exacerbation (7 and 8 weeks respectively). Seemungal and coworkers [8,9] have previously investigated the course of an exacerbation in a cohort of 101 patients with COPD, who had a similar disease severity as our group of 373 patients. They focused on the change in lung function (PEF, FEV_1_ and FVC) and found that there was only a limited fall in lung function during an exacerbation, compatible with our findings. Additionally, they followed the course of an exacerbation with diary cards and reported that the median time to recovery of symptoms was 7 days with an interquartile range of 4 to 14 days. However, the study did not report on the course of individual symptoms. In that respect our findings are new and show that some symptoms remit more rapidly than others. Nocturnal awakening due to respiratory symptoms was especially lingering on for many weeks after an exacerbation. This is in agreement with the findings by Partridge and colleagues that COPD patients indicate the morning as the time when their symptoms are most severe [15]. Whether this reflects some level of cardiac insufficiency is not yet clear. Alternatively, it might reflect a worse impact of ongoing inflammation in the airways at the time that the autonomic nervous imbalance is largest [16]. This clearly needs further study.

Interestingly, the mean course of the increase and decrease in symptoms around the onset of a moderate exacerbation was very similar at the first and the second exacerbation. It thus appears that, despite a large inter-individual difference in the course of an exacerbation, the overall course of a first and second moderate exacerbation is quite similar in one and the same individual. As one can anticipate, the mean values of symptom scores were higher in the period before a severe exacerbation than before the first and second moderate exacerbations. Similarly, salbutamol use was significantly higher in the period before onset of a severe exacerbation and PEF values were significantly lower. Unfortunately, we had insufficient numbers of patients to compare the course of a severe and moderate exacerbation within the same individuals. It remains to be clarified which factors determine a severe exacerbation that requires hospitalization and one that can be managed in the home situation. It may well be, as Donaldson and coworkers suggested [2] that severe exacerbations occur rather in patients with more longstanding COPD, hence the low numbers of patients with both a severe and moderate exacerbation in our study.

We sought for factors in diary cards that would predict an exacerbation in the near future. With each increase in total and separate symptom scores or salbutamol use per week, or decrease in PEF the risk of an exacerbation increased. Although the relative risks were high with weekly increases in reports, the overall predictive value of these changes was still rather limited in view of the moderate values of areas under the ROC curves. Whichever cut-off level one chooses for the increase of the various parameters, the rate of false positive findings remains high and therefore the specificity is low for acceptable values of the sensitivity (Figure 5). The latter suggests that writing down symptoms, salbutamol use or PEF values in reports on a daily basis does not help to assess the risk for an exacerbation on an individual basis.

A study that divided patients into frequent and infrequent exacerbators showed that those with frequent exacerbations had a faster decline in FEV_1_[2]. Moreover, frequent exacerbations are associated with reduced quality of life and daily activities [8,17]. In light of the effect of frequent exacerbations on progression of COPD, our findings also emphasize the importance of preventing COPD exacerbations and particularly to detect patients who are at risk of frequent exacerbations in order to open avenues for reduction in disease progression. Thus, we tried to assess which factors predict that an individual is prone to frequent exacerbations of COPD. As expected, we found a lower FEV_1_ %predicted to be associated with an increased number of exacerbations, a finding compatible with data in the literature [2,17]. Interestingly, and a new observation, the control of COPD questionnaire was associated with the number of exacerbations in patients with moderate to severe COPD as well. In particular, a worse functional status of this domain was associated with frequent exacerbations.

There are some limitations to our study. First, there were relatively few severe exacerbations, probably reflecting the fact that patients were treated and followed accurately in a randomized controlled trial which may have influenced our results. In addition, we cannot rule out the possibility that regular treatment with salmeterol with or without added fluticasone, may have blunted the changes in PEF which recovered very quickly after a COPD exacerbation, whereas this as not the case for the perception of symptoms.

In conclusion, this prospective study in moderate to severe COPD patients shows that symptoms persist for several weeks after an exacerbation, suggesting that the underlying pathophysiology is not resolved with a two-week course of oral corticosteroids and/or antibiotics. This may have implications for a better understanding why exacerbations, which may have a systemic effect on COPD as well, may affect the outcome of COPD. In addition, we show that the course of symptoms is very similar around a first and second exacerbation. Finally, COPD exacerbations can be predicted by an increase in respiratory symptoms, salbutamol use and decrease in PEF, yet the predictive values of these parameters are too low to warrant their daily use in clinical practice.

## Abbreviations

AUC: Area under the curve; COPD: Chronic obstructive pulmonary disease; CCQ: Control of COPD questionnaire; PEF: Peak expiratory flow; FEV_1_: Forced expiratory volume in one second; FVC: Forced vital capacity; GOLD: Global obstructive lung disease; COSMIC: COPD and Seretide: a Multi-Center Intervention and Characterization; ROC: Receiver operating curve.

## Competing interest

M. van den Berge has received research grants from GlaxoSmithKline, E.F.M. Wouters, D.S. Postma and T van der Molen have been consultants for, and received research grants from, GlaxoSmithKline. W.C.J. Hop is a regular statistical consultant for GlaxoSmithKline. J.A. van Noord, J.P.H.M. Creemers, A.J.M. Schreurs received research grants from GlaxoSmithKline.

## Authors’ contributions

MvdB reviewed and interpreted the results and wrote the manuscript. EFMW and DSP designed the study, analyzed the data, reviewed and interpreted the results and wrote the manuscript. WCJH performed the statistical analysis of the data and wrote the manuscript. JAvN, JPHMC and AJMS reviewed and interpreted the results. ThvdM reviewed and interpreted the results and wrote the manuscript. All authors have access to all data in the study and held final responsibility for the decision to submit for publication. All authors read and approved the final manuscript.

## Authors’ information

COSMIC investigators

Dr. R. Aalbers, Dr. F. Beaumont, Dr. W. Boersma, Prof. Dr. J. Bogaard, M. Bunnik, J. Creemers, W. Dalinghaus, C. de Graaff, Dr. J. de Jong, Dr. P. de Jong, D. de Munck, D. de Vries, I. de Vries, W. den Hertog, H. Dik, E. Dubois, M. Eland, W. Evers, S. Gans, W. Geraedts, Dr. H. Heijerman, A. Hendriks, Dr. Ho, Dr. B. Hol, J. Kersbergen, H. Los, P. Luursema, B. Pannekoek, Dr. W. Pieters, R. Quanjel, E. Quanjel-Wisselo, R. Rammeloo, J. Retera, Dr. A. Roldaan, Dr. A. Rudolphus, L. Sala, N. Schlösser, Dr. A. Schols, Dr. A. Schreurs, Dr. J. Simons, A. Sips, Dr. F. Smeenk, W. Strankinga, I. Utama, F. van Beek, H. van de Woude, P. van den Berg, Dr. J. van den Bosch, J. van den Bosch, W. van der Brink, Dr. F. van den Elshout, Dr. B. van der Bruggen- Bogaarts, J. van der Zeijden, A. van Harreveld, Dr. A. van Keimpema, Dr. J. van Noord, H. van Pagée, R. van Snippenburg, P. van Spiegel, Dr. J. Verbraecken, J. Westbroek; The Netherlands.

## How does this advance the field?

This study shows that COPD exacerbations can be predicted by an increase in respiratory symptoms, salbutamol use and decrease in Peak Expiratory Flow (PEF) values, but the predictive value of these parameters is low. Remarkably, symptoms persist for up to 8 weeks after a COPD exacerbation. Whether this reflects incomplete resolution of the pathophysiology underlying exacerbations remains to be elucidated.

## Role of the funding source

The study sponsor, GlaxoSmithKline, was involved in the study design, together with the principal investigators, in the collection and analysis of data, which were made freely available to the investigators, and in the decision to submit the paper for publication.
